# Psychological Alterations in Youths with Type I Diabetes: Associations with Salivary Cortisol Concentration

**DOI:** 10.3390/medicina60010019

**Published:** 2023-12-21

**Authors:** Nisrin El Mlili, Hanan Ahabrach, Hind Bahri, Abdelilah Kerkeb, Mayra Alejandra Mafla-España, Omar Cauli

**Affiliations:** 1 Institute of Nursing and Health Technology (ISPITS), Tetouan 93000, Morocco; bioniss@hotmail.com (N.E.M.); hananahabrach@yahoo.fr (H.A.); hind.bahri@etu.uae.ac.ma (H.B.); 2 Department of Biology and Health, Faculty of Sciences, Abdelmalek Essaâdi University, Tetouan 93000, Morocco; 3 Interdisciplinary Laboratory for Research in Pedagogical Engineering (LIRIP), Ecole Normale Supérieure, Abdelmalek Essaâdi University, Tetouan 93000, Morocco; 4 Koelma Urban Health Center, Tetouan 93000, Morocco; kerkebabdelilah@hotmail.com; 5 Department of Nursing, University of Valencia, 46010 Valencia, Spain; maymaes@alumni.uv.es

**Keywords:** anxiety, attention deficit hyperactivity disorder, sleep, stress, glycaemia-, children

## Abstract

*Background and Objectives:* Type 1 diabetes mellitus (T1DM) is one of the most common chronic diseases in children and adolescents, and is associated with stress and other psychological alterations. This study aims to assess psychological and sleep disorders and health-related quality of life in young people with T1DM and to determine the relationship between these parameters and levels of salivary cortisol, a hormone widely associated with stress and several psychological symptoms. *Materials and Methods:* In our cross-sectional study performed in 60 Moroccan children and adolescents with T1DM, detailed psychological evaluations were performed to assess symptoms of anxiety, attention-deficit hyperactivity disorder (ADHD), sleep quality and diabetes-specific quality of life (using the RCMAS-2, ADHD rating scale, Pittsburgh scale and the DQoL scale, respectively), and cortisol concentration was measured from saliva samples taken mid-morning. *Results:* A total of 60 children and adolescents with T1DM were recruited. The mean age was 11.05 ± 0.35 (6–17). The mean salivary cortisol level in ng/mL was 4.7 ± 0.49 (0.7–20.2) and was significantly associated with an anxiety RCMAS2 score for the Worry subdomain and DQoL subdomain “Anxiety”. Linear regression analysis showed that salivary cortisol was significantly higher in girls compared to boys (*p* = 0.004) (beta coefficient: 3.384 CI95%: 1.137–5.630) and with Hb1AC level as a continuous variable (*p* = 0.0001) (beta coefficient: 1.135 CI95%: 0.509–1.760). The other variables included in the model were not significant (*p* > 0.05). There was an association between salivary cortisol concentration with anxiety RCMAS2 score for Worry subdomain and QoL sub-domain “Anxiety”. Still, a significant (*p* = 0.018) association emerged for anxiety RCMAS2 score Worry subdomain and QoL anxiety subdomain (*p* = 0.044). *Conclusions:* Children and adolescents with T1DM experienced significantly elevated symptoms of anxiety and sleep disturbances, particularly in girls, and frequent symptoms of ADHD, particularly in boys. Salivary cortisol concentration collected in the morning is associated with anxiety burden but not with other psychological alterations. Further studies are needed to clarify the associations between salivary cortisol concentration and anxiety in type 1 diabetes in order to propose the hormone as a biomarker for interventions aimed to reduce anxiety levels in these patients.

## 1. Introduction

Type 1 diabetes mellitus (T1DM) is one of the most common chronic diseases in children/adolescents, with about 1.5 million of the 8.4 million individuals worldwide diagnosed with T1DM in 2021 being less than 20 years old [1].

The chronic nature of the disease and the constraints related to treatment and follow-up expose children and adolescents with diabetes to a higher risk of developing psychological disorders compared to their healthy peers. The higher risk of developing psychological disorders for T1DM patients is not only due to the chronic nature of the disease and issues related to treatment, but can be also due to glucose-level variations, insulin deprivation and other mechanisms that have direct effects on brain functioning, and therefore on the risk of developing psychological disorders [2].

Symptoms of depression, anxiety and irritability are most frequently reported in people with diabetes [3]. Some studies show that higher levels of state anxiety symptoms are associated with less frequent blood glucose monitoring and suboptimal glycemic control [4]. Furthermore, children with diabetes suffer from ADHD much more frequently than those without diabetes [5]. A recent meta-analysis confirmed the bidirectional associations between ADHD and T1DM [6].

Young people with diabetes are also prone to sleep disturbances. Studies carried out in young people with T1DM have shown that they have a short sleep duration [7,8] and reduced slow-wave sleep compared to their non-diabetic peers [9]. Reduced slow-wave sleep is associated with reduced self-management of diabetes, poor glycemic control, greater daytime sleepiness and poor quality of life [10]. Nonetheless, a recent study [11] carried out in a group of adolescents with T1DM and their peers showed that the prevalence of sleep disorders in T1DM patients was not higher than in the non-diabetic population, but girls in the latter group had significantly poor sleep quality. The sleep disturbances and the development of psychosocial illness in diabetic children can lead to non-adherence to treatment, increased risk of complications and deterioration in the quality of life [12].

Cortisol seems to be one of the most widely used salivary markers when evaluating psychological alterations in people with diabetes [13]. Cortisol is the end product of the hypothalamic–pituitary–adrenal (HPA) axis, and mainly secreted as a reaction to stress. It plays an essential role in the normal physiology of the organism [14,15,16]. Alterations in the HPA axis and cortisol concentrations have been linked to the pathophysiology of a number of diseases, including diabetes [17,18,19,20,21]. Other studies found altered activity of the HPA axis in T1DM patients, but there were no significant differences in the mean nocturnal cortisol values or peak daytime cortisol levels between healthy controls and patients with T1DM [22].

Psychosocial factors play a role both in the etiopathogenesis of diabetes mellitus and in the management of the disease [23]. However, although the physiological complications of diabetes are generally taken into consideration by medical staff, the psychosocial aspects remain largely neglected. Focusing on the psychological alterations and integrating them into the management of diabetes should therefore be a major concern for the practitioner responsible for treating the disease, since diabetes remains a debilitating and fatal condition in developing countries [24].

This study aims to assess whether prevalent psychological alterations found in youth with diabetes type 1 such as specific anxiety symptoms, attention-deficit hyperactivity disorder (ADHD) symptoms, sleep quality, and health-related quality of life are associated with morning salivary cortisol level. Multivariate analyses were performed to evaluate the influence of demographic and clinical factors affecting cortisol levels such as age, gender, pubertal status, duration of the disease, body mass index, glycemic control or familial history of diabetes.

## 2. Methods

### 2.1. Participants

Children and adolescents aged between 6 and 17 years, diagnosed with T1DM for at least 1 year prior to the beginning of the study, were recruited for this study. The sample consisted of 60 children and adolescents with T1DM who met the eligibility criteria for the study (more than 1 year since diagnosis of T1DM, treated with insulin and not suffering from another kind of diabetes or another condition that affects health and prevents comprehension of the questionnaires). Using a demographic data sheet, we obtained information from all the participants, including sex, age, educational level, date of diabetes diagnosis, family socioeconomic status and history of type 1 or 2 diabetes. Information about glycated hemoglobin (HbA1c) for the three months prior to the study was also collected. All participants were receiving insulin treatment. None had any diabetes-related complications. The information of children younger than 16 years was obtained by their parents, who answered a self-administered questionnaire relating to their children’s psychological characteristics and their diabetes-related quality of life.

The study protocol was approved by the Biomedical Research Ethics Committee of the University of Oujda, Morocco (CERBO 06/2022). Data collection was carried out between November 2021 and June 2022. The research and the informed consent were presented to the children’s/adolescents’ guardians, and the informed assent form to children and adolescents. After they agreed to participate, the data were collected and the study questionnaires were applied.

### 2.2. Psychological Assessments

The Pittsburgh Sleep Quality Index (PSQI), Attention-Deficit Hyperactivity Disorder (ADHD), the Revised Children’s Manifest Anxiety Scale—Second Edition (RCMAS-2) and the Diabetes Quality of Life questionnaire (DQoL) were completed to define the presence and severity of sleep quality, ADHD symptoms, anxiety, and quality of life, respectively. Sleep quality was assessed using a validated Arabic language version of the PSQI [25]. The PSQI is a self-administered questionnaire [26] that takes approximately 5–10 min to complete. It consists of 19 items that cover seven major issues: subjective sleep quality, sleep onset latency, total sleep duration, sleep efficiency, sleep disturbances, use of sleep medication, and daytime dysfunction. Scoring is based on a scale from 0 to 3 for each item. PSQI global scores range from 0 to 21, while a score higher than 5 indicates sleep disorders.

The Attention Deficit Hyperactivity Disorder scale was used to measure the deficit of attention and hyperactivity. We used the Arabic version of the ADHD Rating Scale [27]. This is the translated and linguistically standardized version of the original ADHD Rating Scale [28]. It consists of 14 items, and each item can be rated on a scale of 0 to 3 arranged in the following format: 0 = not at all, 1 = just a little, 2 = pretty much and 3 = very much. The questionnaire takes approximately 5 min to complete. Three scores are possible after administering the scale: (a) the grand total score, (b) the inattention–restlessness subscale score (including items aimed at identifying symptoms thought to be relevant to the inattention–restlessness factor), and (c) the impulsivity–hyperactivity sub-scale score (including items to test for symptoms that are thought to be explicitly related to the impulsivity/hyperactivity factor).

The Arabic version of the Revised Children’s Manifest Anxiety Scale—Second Edition (RCMAS-2) was used to assess anxiety [29]. The RCMAS-2 is a self-report measure designed to measure the level and nature of anxiety experienced by children and adolescents between 6 and 19 years old [30]. The respondents are asked to read each item on the scale and indicate their response using a dichotomized format (yes = 1 and no = 0). It consists of 49 items and includes three subscales (Physiological Anxiety, Worry, and Social Anxiety) as well as a total anxiety score. In addition, the RCMAS-2 has two validity indices. The score on the RCMAS-2 scale represents the sum of the results on the whole RCMAS-2 scale. Higher scores (scores above 60) indicate the respondent may have anxiety.

The Arabic version of the Diabetes Quality of Life questionnaire [31] was used to assess the impact of intensive insulin treatment on the lives of people with type 1 diabetes. The DQoL questionnaire [32] is composed of 46 items divided into 3 dimensions: life satisfaction (15 items), impact of diabetes (20 items) and worries about the future effects of diabetes (11 items divided into social/vocational and diabetes-related). Each item can be given 1–5 points on a Likert scale. Satisfaction is rated from very satisfied (1 point) to very dissatisfied (5 points). The Impact and Worry subscales are rated from no impact or never worried (1 point) to always impacted or worried (5 points).

### 2.3. Salivary Cortisol Concentration

Specimens were collected on two separate days (two samples for each subject) by rolling the cylindrical absorbent cotton swab (Salivette^®^, SARSTEDT, Numbrecht, Germany) in the mouth for approximately 2 to 3 min before replacing it in the collection tube and capping it tightly. The collected samples had to be stored in the freezer at −20 °C. The samples were thawed and centrifuged at 10,000 rpm for 5 min, then the saliva was extracted, and the samples were placed in Eppendorf microtubes. Aliquots of 100 µL of salivary sample from each participant were transferred to microtubes and 120 µL of ethyl acetate was added to each salivary sample and labeled, respectively. The samples were centrifuged again at 10,000 rpm for 10 min and 0.80 of the ethyl acetate extract was extracted and placed in new Eppendorf tubes. Then, 5 µL was injected into the LC-MS/MS using an ExionLC AD coupled to a Sciex QTRAP 6500+ mass spectrometer. The HPLC column was C18 BEH, 50 × 2.1 mm × 1.7 U (Waters). Chromatography was performed in a 10 min run with two phases: A: deionized water (2 mM ammonium acetate + 0.1%) formic acid and B: methanol (2 mM ammonium acetate + 0.1%) formic acid. %). The intra-assay and inter-assay coefficients of variation were <4%.

### 2.4. Statistical Analysis

A descriptive analysis of the sociodemographic variables was performed, showing the distribution of percentages for the categorical variables, and the mean and standard error of the mean for the quantitative variables. For the bivariate analysis, the normal distribution of each variable was first calculated using the Kolmogorov–Smirnov test. The correlation between quantitative variables was determined using the Spearman correlation test (non-parametric test) or the Pearson correlation test (parametric test). Non-parametric Mann–Whitney U tests were used for to compare means between the quantitative variables, since not all of the variables presented a normal distribution, and the Chi-square test was used for the comparison of proportions. We used linear regression models to examine the association of morning cortisol concentration with age, gender, puberty status, familiar history of diabetes, glycemic control (as a continuous and categorical variable based on 7.5% cut-off) and BMI (as a continuous and categorical variable) in a multivariable linear regression model. The statistical significance was set at *p* < 0.05. All statistical analyses were performed using the SPSS statistical package (version 28.0; SPSS, Inc., Chicago, IL, USA).

## 3. Results

### 3.1. Sociodemographic and Clinical Data

A total of 60 children and adolescents diagnosed with type I diabetes mellitus with a mean age of 11.05 ± 0.35 (SEM) (range 6–17 years) were included in this study. Most of the participants were girls (75%) and 25% were boys; 58.1% of the participants had a family member with type I diabetes mellitus, while 40.3% did not. Regarding BMI, according to the age and sex of each participant, 67.7% had a normal weight, 21% were overweight, 1.6% obesity, and 4.8% were under weight (Table 1).

### 3.2. Psychological and Diabetes-Related Quality of Life Assessments

The mean value in the Pittsburgh Sleep Quality Index was 4.02 ± 0.25 (SEM) (range 0–8 points). The mean value on the ADHD scale was 15.1 ± 1.09 (SEM) (range 1–35 points). The mean value on the RCMAS-2 child anxiety scale was 16.2 ± 0.97 (SEM) (range 2–33 points) and the mean value on the QOL scale was 86.5 ± 1.43 (SEM) (range 62–115 points).

Each variable on the psychological scales was dichotomized considering their respective cut-off (1 = when the score of each scale indicates the presence of a disorder and 0 = when the score of each scale is normal). It was also dichotomized as the presence or absence of a psychological problem considering the lowest or highest quartiles for each scale analyzed.

According to the cut-off (≥5 points) for the Pittsburgh scale [26], 44.4% of girls and 33.3% of boys met the sleep disability criteria. In addition, according to the cut-off (≥21 points) of the RCMAS-2 scale [30], 26.6% of girls and 20% of boys presented anxiety symptoms. Meanwhile, 11.1% of girls had a score >23.5 (cut-off) on the ADHD scale, while the same was true for 40% of boys.

**Figure 1:**
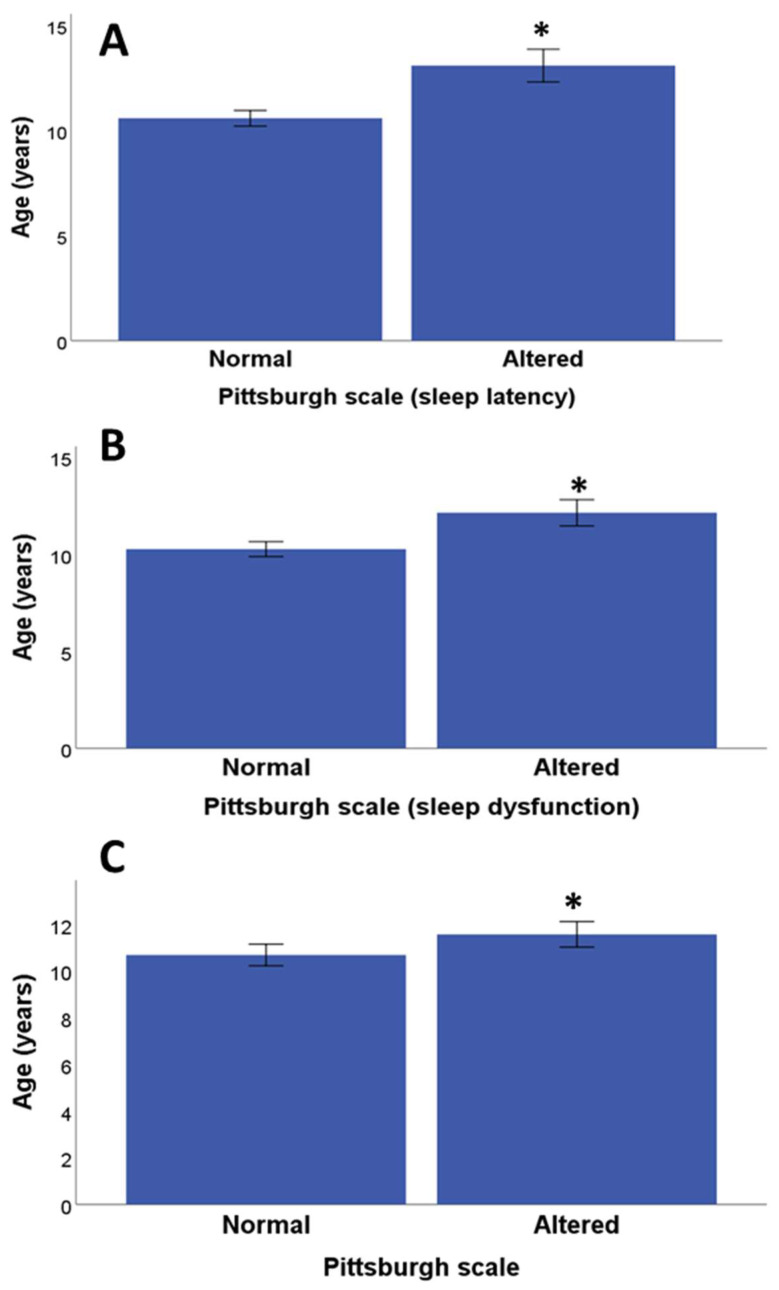
Age differences and sleep quality. (**A**) Age differences between children/adolescents with normal and altered Pittsburg subscale sleep latency; (**B**) age differences between children/adolescents with normal and altered the Pittsburg subscale sleep dysfunction; (**C**) age differences between children/adolescents with normal and altered sleep quality based on cut-off of Pittsburg scale. * *p* < 0.05 normal versus altered.

Correlations were performed to see if there were possible associations between age and the scores of the psychological scales. Significant associations were found between the Pittsburgh subscales and age: sleep latency (Rho = 0.33, *p* = 0.009, Pearson correlation test) (Figure 1A), between age and sleep dysfunction (Rho = 0.38, *p* = 0.002, Pearson correlation test) (Figure 1B) and between age and the Pittsburgh Scale total score (Rho = 0.27, *p* = 0.03, Pearson correlation test) (Figure 1C), while there were no significant associations with the rest of the subscales of subjective sleep quality (Rho = 0.06, *p* = 0.63, Pearson correlation test), sleep duration (Rho = 0.10, *p* = 0.44, Pearson correlation test), sleep efficiency (Rho = −0.15, *p* = 0.26, Pearson correlation test), sleep disturbance (Rho = 0.09, *p* = 0.48, Pearson correlation test) or medication use (Rho = −0.14, *p* = 0.27, Pearson correlation test).

Similarly, no significant associations were found between the ADHD scale and age (Rho = 0.06, *p* = 0.61, Pearson correlation test), or between age and the RMCAS-2 anxiety scale and its subscales: physiological anxiety (Rho = 0.13, *p* = 0.30, Pearson correlation test), worry (Rho = −0.06, *p* = 0.63, Pearson correlation test), social anxiety (Rho = −0.11, *p* = 0.38, Pearson correlation test) and RCMAS-2 total score (Rho = −0.03, *p* = 0.77, Pearson correlation test). There was a significant association between the subscales of quality of life and age, QOL satisfaction (Rho = −0.26, *p* = 0.04, Pearson correlation test), QOL impact (Rho = 0.35, *p* = 0.007, Pearson correlation test). No significant associations were found for age and QOL anxiety (Rho = 0.04, *p* = 0.73, Pearson correlation test), and for age and QOL score there were no significant associations (Rho = −0.01, *p* = 0.93, Pearson correlation test).

No significant difference was found between sex and the Pittsburgh scale total score (*p* = 0.42, Mann–Whitney U test), while significant differences were found between sex and the Pittsburgh sleep disorders subscale and sex (*p* = 0.02, Mann–Whitney U test). There was no significant difference between gender and the ADHD scale (*p* = 0.07, Mann–Whitney U test). Likewise, there were no significant differences between sexes for the RCMAS-2 total score (*p* = 0.61, Mann–Whitney U test) or the total QOL score (*p* = 0.06, Mann–Whitney U test).

### 3.3. Relationship between Salivary Cortisol Values and Psychological Assessment

**Figure 2:**
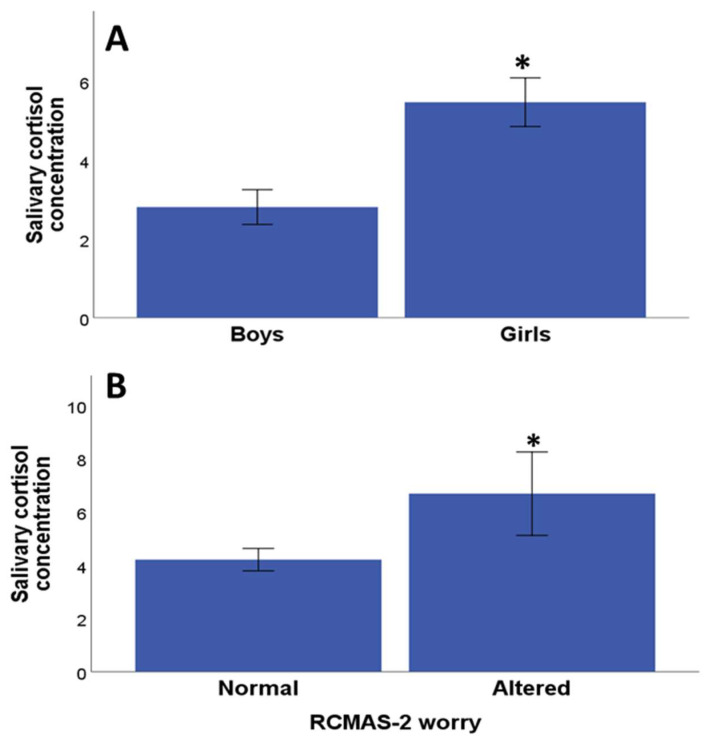
Associations between salivary cortisol concentration and gender and psychological symptoms. (**A**) Differences in salivary cortisol concentration between boys and girls; (**B**) differences in salivary cortisol concentration between children/adolescents with normal and altered “worry” subscale of RCMAS-2. * *p* < 0.05 normal versus altered.

Significant differences were found between salivary cortisol concentrations between genders, with girls showing a higher concentration of cortisol compared with boys (*p* = 0.008 Mann–Whitney U test) (Figure 2A). There was no significant association between salivary cortisol concentration and age (Rho = −0.01, *p* = 0.90, Pearson correlation test). There was no significant difference between salivary cortisol concentration and the Pittsburgh scale total score (*p* = 0.82, Mann–Whitney U test), and likewise, there were no significant differences between the salivary cortisol concentration and the Pittsburgh subscales sleep quality (*p* = 0.18, Mann–Whitney U test), sleep latency (*p* = 0.24, Mann–Whitney U test), sleep duration (*p* = 0.94, Mann–Whitney U), sleep efficiency (*p* = 0.73, Mann–Whitney U test), sleep disturbance (*p* = 0.21, Mann–Whitney U test), medication use (*p* = 0, 96, Mann–Whitney U test) and sleep dysfunction (*p* = 0.92, Mann–Whitney U test). Likewise, there were no significant differences between salivary cortisol concentration and the ADHD scale (*p* = 0.39, Mann–Whitney U test), between salivary cortisol concentration and the RMCAS-2 total score scale (*p* = 0.30, Mann–Whitney U test) or its subscales: physiological anxiety (*p* = 0.58, Mann–Whitney U test). A significant difference was found between the salivary cortisol concentration with the worry subscale (*p* = 0.02, Mann–Whitney U test) (Figure 2B), while with the other subscales, no significant differences were found between salivary cortisol concentration and social anxiety (*p* = 0.25, Mann–Whitney U test). Similarly, there were no significant differences between salivary cortisol concentration and the quality-of-life subscales: satisfaction with quality of life (*p* = 0.71, Mann–Whitney U test), impact on quality of life (*p* = 0.24, Mann–Whitney U test), and quality of life anxiety (*p* = 1.00, Mann–Whitney U test).

Stepwise multiple linear regression analysis was used to investigate independent predictors of salivary cortisol concentration with scores in the instrument aiming to quantify psychological variables and quality of life assessment. The two psychological assessments that were significantly associated with salivary cortisol concentration were RCMAS2 score for the Worry subdomain (*p* = 0.02) and the QoL subdomain “Anxiety” (*p* = 0.043) (Table 2).

### 3.4. Relationship between Clinical Parameters and Cortisol Concentration

Next, we evaluated whether age, gender, puberty status, familial history of diabetes, glycemic control (as a continuous and categorical variable based on 7.5% cut-off) and BMI (as a continuous and categorical variable) were associated with salivary cortisol concentration in a multivariable linear regression model. Gender was significantly associated with a high cortisol concentration in girls (*p* = 0.004) (beta coefficient: 3.384 CI95%:1.137–5.630) and Hb1AC as a continuous variable (*p* = 0.0001) (beta coefficient: 1.135 CI95%:0.509–1.760). The other variables included in the model were not significant (*p* > 0.05). Based on these results, we analyzed whether gender and Hb1AC mediate the effect observed between the association between salivary cortisol concentration with anxiety RCMAS2 score for Worry subdomain and QoL subdomain “Anxiety”. Still, a significant (*p* = 0.018) association emerged for the anxiety RCMAS2 score Worry subdomain and QoL anxiety subdomain (*p* = 0.044).

## 4. Discussion

One of the main findings of this cross-sectional study was that 44.4% of the girls and 33.3% of the boys met the sleep disability criteria. We found that 26.6% of the girls and 20% of the boys presented anxiety symptoms, while 11.1% of the girls and 40% of the boys presented high scores on the ADHD scale. These psychological alterations were associated with a poor diabetes-related quality of life. The high prevalence of psychological alterations reported in our study indicate that the children and adolescents should be referred to specialists to receive proper management; as is the case for adults with diabetes, healthcare professionals should take into account not only the physical effects of the disease, but also the mental health problems associated with it [33,34]. HbA1c is a biomarker with a central role in the diagnosis and follow-up of patients with diabetes. Several studies have linked poor glycemic control to psychological and mental health issues [35,36,37,38,39,40,41,42,43]. However, the findings of these studies are contradictory, with some of them only showing a weak association, or no association at all, between health problems and glycemic control [36,44,45,46]. Previous studies reported that there was a relationship between HbA1C and quality of life of children with TIDM, where children who achieved the HbA1C target of <7.5% reported higher quality of life; HbA1C > 7.5 and <9.0% reported significantly lower quality of life; the worst control with HbA1C > 9.0% reported significantly lower quality of life than each of the other two HbA1C groups [47]. However, in our study, we did not observe any significant association between glycemic control and other clinical variables (body-mass index, family history of diabetes, number of years since diagnosis) and psychological assessments or with diabetes-related quality of life. Social and family factors should be involved in the psychological alterations [33,48,49] rather a glycemic control, as demonstrated by the fact type 1 diabetic adolescents who had better metabolic control reported more conflict regarding family relationships and issues of independence than did those with lesser control [50]. Adolescents with diabetes who perceived they had more social support experienced better control of their diabetes. The more disengaged the family system, the worse the diabetic control of the adolescent [51].

It has been well documented that in addition to chronic hyperglycemia and relative insulin insufficiency, the activity of the HPA axis is altered in type I diabetic adults [52] and children [53]. HPA axis activity is intimately related to chronic stress and anxiety [54,55], and is associated with several pathological situations including diabetes [52]. One of the most widely used markers for examining HPA axis functioning is cortisol concentration [56], and in particular, salivary cortisol concentration has been proposed as a suitable, non-invasive biomarker to detect psychological alterations [53,57].

In our study, significantly higher levels of salivary cortisol concentration were found in girls in comparison with boys. Sex differences in salivary cortisol response to stressors have been widely documented [58]. Since diabetes management and glycemic control are common causes of stress, the increase in cortisol concentration may be due to hyperactivity of the HPA axis as a result of stress caused by conditions related to the pathology of type 1 diabetes, particularly in girls [57]. A recent study revealed subtle normative sex differences in the influence of pubertal maturation on HPA regulation at the pituitary level. This normative shift may tip the balance towards stress response dysregulation in girls at high risk for anxiety and depression and may represent one potential mechanism underlying elevated rates of depression among pubescent girls [59]. This gender differences may suggest that girls could benefit more from psychological intervention aimed at reducing or managing anxiety symptoms, as these have detrimental effects on health outcomes, including self-management, quality of life, and HbA1c [35].

We examined anxiety using a validated version of the RCMAC-2 scale that measures the level and nature of anxiety. We found a significant correlation between salivary cortisol concentration and the worry subscale of RMCAS-2 in children with T1DM. Anxiety symptoms are considered the most prevalent mental health condition in young people with T1D (see [35] for a review). Low blood sugar and anxiety are linked, but the relationship is complicated. Symptoms of low blood sugar can mirror anxiety’s symptoms, or worsen existing anxiety [4,60]. These patients may also experience anxiety about regulating their blood sugar levels. This may manifest as a consistent and overwhelming fear of becoming hypoglycemic or anxiety about managing diabetes [61]. However, studies examining the relationship between salivary or blood cortisol and anxiety level are very scarce even in children and adolescents without diabetes. The authors of [53] measured salivary cortisol and anxiety using the State-Trait Anxiety (STAI) Inventory in prepubertal patients (aged 6–12 years) with T1DM and their control peers. They found no difference in salivary cortisol levels between the T1DM patient group and the control group or with anxiety level. However, it should be taken into account that in the previous study, the mean level of glycemic control (Hb1Ac mean 7.6%) was better compared to that of our study (Hb1AC mean 9%) and, as demonstrated in our study, there is a correlation between salivary cortisol concentration and blood Hb1AC levels which could explain the discrepancy beside the fact that another anxiety scale was used in our study. As a limitation in our study, we did not compare the outcomes with control group of children and adolescents without diabetes or with another chronic condition in order to assess if salivary cortisol and anxiety are related in a broader sense or more limited to the diabetes context. The fact that cortisol level in saliva, a non-invasive and easily collectable biological fluid, is associated with anxiety opens up the possibility of providing an objective biomarker useful, beside the psychological evaluation, for studying anxiety in type 1 diabetes patients. The next step is to analyze whether this biomarker could change after interventions aimed to reduce anxiety in these patients.

## 5. Conclusions

Children and adolescents with T1D presented several psychological alterations such as sleep disturbances, anxiety and ADHD symptoms, and a poor quality of life. Salivary cortisol concentration correlated with some aspects of anxiety, such as the RMCAS-2 worry subscale, particularly in female individuals. There is a need for further research to clarify the association between cortisol concentration and psychological disturbances in type 1 diabetes in longitudinal studies with a gender-specific analysis.

## Figures and Tables

**Table 1:** Sociodemographic and clinical characteristics.

Variables	Mean and Standard Error of the Mean (Min-Max Range) (Discrete Variables) or Frequencies (Categorical Variables)
Age	11.05 ± 0.35 (6–17)
Puberty	Pre-pubertal:58.3% (*n* = 35) Post-pubertal: 41.7% (*n* = 25)
Number of years elapsed since diabetes diagnosis	3.40 ± 0.26 (1–9)
BMI	18.5 ± 0.31 (13.9–25.8 kg/m^2^)
BMI categories	Normal weight 71.6% (*n* = 43) Underweight 5.0% (*n* = 3) Overweight 23.4% (*n* = 14)
Mean concentration of cortisol in saliva ng/mL	4.77 ± 0.49 (0.7–20.2)
Mean concentration of H1bAC	9.0 ± 0.25 (5.5–15)
Glycemic control	Good (Hb1AC < 7.5%): 21.7% (*n* = 13) Poor (Hb1AC ≥ 7.5%): 78.3% (*n* = 47)
Family history of diabetes (type 1 and 2) (first-degree relatives)	Yes: 58.3% (*n* = 35) No:41.7% (*n* = 25)

**Table 2:** The association of salivary cortisol concentration with score in the instrument aimed to quantify psychological variables and quality of life assessment. Multivariable linear regression model.

Variable	Standard B Coefficient	t	*p* Value	95% Confidence Interval Lower Limit Upper Limit	
ADHD score	−0.285	−1.713	0.096	−0.268	0.023
Pittsburg index for “Sleep quality”	−0.043	−0.274	0.786	−1.265	0.965
Pittsburg index for “Sleep latency”	−0.305	−1.486	0.147	−3.106	0.486
Pittsburg index for “Sleep duration”	−0.017	−0.112	0.912	−8.434	7.556
Pittsburg index for “Sleep efficiency”	0.071	0.434	0.668	−6.727	10.364
Pittsburg index for “Sleep disturbances”	0.053	0.321	0.750	−1.845	2.535
Pittsburg index for “Use of hypnotic medication”	−0.071	−0.441	0.662	−2.423	1.561
Pittsburg index for “Daytime dysfunction”	0.197	1.096	0.281	−0.920	3.064
RCMAS2 score for “Physiological anxiety”	0.160	0.824	0.416	−0.353	0.833
RCMAS2 score for Worry	0.537	2.117	0.042 *	0.021	1.092
RCMAS2 score for social anxiety	0.009	0.046	0.964	−0.440	0.460
QoL subdomain “Impact”	−0.017	−0.087	0.931	−0.246	0.226
QoL subdomain “Anxiety”	0.392	1.851	0.043 *	0.017	0.766
QoL total score	0.056	0.276	0.784	−0.122	0.160

Footnote: * means significant values.

## Data Availability

The data presented in this study are available on request with scientific purpose from the corresponding author.

## References

[1] Gregory G.A., Robinson T.I.G., Linklater S.E., Wang F., Colagiuri S., de Beaufort C., Donaghue K.C., Magliano D.J., Maniam J., Orchard T.J. (2022). Global Incidence, Prevalence, and Mortality of Type 1 Diabetes in 2021 with Projection to 2040: A Modelling Study. Lancet Diabetes Endocrinol..

[2] Barat P., Brossaud J., Lacoste A., Vautier V., Nacka F., Moisan M.-P., Corcuff J.-B. (2013). Nocturnal Activity of 11β-Hydroxy Steroid Dehydrogenase Type 1 Is Increased in Type 1 Diabetic Children. Diabetes Metab..

[3] DiMeglio L.A., Acerini C.L., Codner E., Craig M.E., Hofer S.E., Pillay K., Maahs D.M. (2018). ISPAD Clinical Practice Consensus Guidelines 2018: Glycemic Control Targets and Glucose Monitoring for Children, Adolescents, and Young Adults with Diabetes. Pediatr. Diabetes.

[4] Herzer M., Hood K.K. (2010). Anxiety Symptoms in Adolescents with Type 1 Diabetes: Association with Blood Glucose Monitoring and Glycemic Control. J. Pediatr. Psychol..

[5] Kapellen T.M., Reimann R., Kiess W., Kostev K. (2016). Prevalence of Medically Treated Children with ADHD and Type 1 Diabetes in Germany—Analysis of Two Representative Databases. J. Pediatr. Endocrinol. Metab..

[6] Ai Y., Zhao J., Liu H., Li J., Zhu T. (2022). The Relationship between Diabetes Mellitus and Attention Deficit Hyperactivity Disorder: A Systematic Review and Meta-Analysis. Front. Pediatr..

[7] Estrada C.L., Danielson K.K., Drum M.L., Lipton R.B. (2012). Insufficient Sleep in Young Patients with Diabetes and Their Families. Biol. Res. Nurs..

[8] Reutrakul S., Thakkinstian A., Anothaisintawee T., Chontong S., Borel A.-L., Perfect M.M., Janovsky C.C.P.S., Kessler R., Schultes B., Harsch I.A. (2016). Sleep Characteristics in Type 1 Diabetes and Associations with Glycemic Control: Systematic Review and Meta-Analysis. Sleep Med..

[9] Perfect M.M., Elkins G.R., Lyle-Lahroud T., Posey J.R. (2010). Stress and Quality of Sleep among Individuals Diagnosed with Diabetes. Stress Health.

[10] McDonough R.J., Clements M.A., DeLurgio S.A., Patton S.R. (2017). Sleep Duration and Its Impact on Adherence in Adolescents with Type 1 Diabetes Mellitus. Pediatr. Diabetes.

[11] Çömlek F., Çelik H., Keskin B., Süt N., Dilek E., Tütüncüler F. (2021). Sleep Quality Assessment in Adolescents with and without Type 1 Diabetes Using the Pittsburg Sleep Quality Index. Indian J. Endocrinol. Metab..

[12] Kakleas K., Kandyla B., Karayianni C., Karavanaki K. (2009). Psychosocial Problems in Adolescents with Type 1 Diabetes Mellitus. Diabetes Metab..

[13] Bargues-Navarro G., Ibáñez-del Valle V., El Mlili N., Cauli O. (2022). Salivary Biomarkers Associated with Psychological Alterations in Patients with Diabetes: A Systematic Review. Medicina.

[14] O’Connor T.M. (2000). The Stress Response and the Hypothalamic-Pituitary-Adrenal Axis: From Molecule to Melancholia. QJM.

[15] Aminkeng F., Ross C.J.D., Rassekh S.R., Hwang S., Rieder M.J., Bhavsar A.P., Smith A., Sanatani S., Gelmon K.A., Bernstein D. (2016). Recommendations for Genetic Testing to Reduce the Incidence of Anthracycline-induced Cardiotoxicity. Br. J. Clin. Pharmacol..

[16] Smith L.K., Cidlowski J.A. (2010). Glucocorticoid-Induced Apoptosis of Healthy and Malignant Lymphocytes. Prog. Brain Res..

[17] Roy M.S., Roy A., Gallucci W.T., Collier B., Young K., Kamilaris T.C., Chrousos G.P. (1993). The Ovine Corticotropin-Releasing Hormone-Stimulation Test in Type I Diabetic Patients and Controls: Suggestion of Mild Chronic Hypercortisolism. Metabolism.

[18] Gaete X., Iñiguez G., Linares J., Avila A., Mericq V. (2013). Cortisol Hyporesponsiveness to the Low Dose ACTH Test Is a Frequent Finding in a Pediatric Population with Type 1 Diabetes Mellitus. Pediatr. Diabetes.

[19] Simunkova K., Hampl R., Hill M., Kriz L., Hrda P., Janickova-Zdarska D., Zamrazil V., Vrbikova J., Vondra K. (2010). Adreno-cortical Function in Young Adults with Diabetes Mellitus Type 1. J. Steroid Biochem. Mol. Biol..

[20] Korczak D.J., Pereira S., Koulajian K., Matejcek A., Giacca A. (2011). Type 1 Diabetes Mellitus and Major Depressive Disorder: Evidence for a Biological Link. Diabetologia.

[21] Lebinger T.G., Saenger P., Fukushima D.K., Kream J., Wu R., Finkelstein J.W. (1983). Twenty-Four-Hour Cortisol Profiles Demonstrate Exaggerated Nocturnal Rise in Diabetic Children. Diabetes Care.

[22] Vanaelst B., De Vriendt T., Huybrechts I., Rinaldi S., De Henauw S. (2012). Epidemiological Approaches to Measure Childhood Stress. Paediatr. Perinat. Epidemiol..

[23] Nygren M., Carstensen J., Koch F., Ludvigsson J., Frostell A. (2015). Experience of a Serious Life Event Increases the Risk for Childhood Type 1 Diabetes: The ABIS Population-Based Prospective Cohort Study. Diabetologia.

[24] Peuhmond A. (2016). CA-163: Vécu de l’enfant et de l’adolescent Diabétique à Propos de 3 Cas Au Centre Anti-Diabétique d’Abidjan: Difficultés et Perspectives. Diabetes Metab..

[25] Suleiman K.H., Yates B.C., Berger A.M., Pozehl B., Meza J. (2010). Translating the Pittsburgh Sleep Quality Index into Arabic. West. J. Nurs. Res..

[26] Buysse D.J., Reynolds C.F., Monk T.H., Berman S.R., Kupfer D.J. (1989). The Pittsburgh Sleep Quality Index: A New Instrument for Psychiatric Practice and Research. Psychiatry Res..

[27] Hassan A.M., Al-Haidar F., Al-Alim F., Al-Hag O. (2009). A Screening Tool for Attention Deficit Hyperactivity Disorder in Children in Saudi Arabia. Ann. Saudi Med..

[28] DuPaul G.J., Anastopoulos A.D., Power T.J., Reid R., Ikeda M.J., McGoey K.E. (1998). Parent Ratings of Atten-tion-Deficit/Hyperactivity Disorder Symptoms: Factor Structure and Normative Data. J. Psychopathol. Behav. Assess..

[29] Al Jabery M.A., Arabiat D.H. (2011). Psychometric Properties of the Arabic Translated Version of the RCMAS: Preliminary Indicators from a Jordanian Sample. J. Int. Couns. Educ..

[30] Reynolds C.R., Richmond B.O. (2008). Revised Children’s Manifest Anxiety Scales-Second Edition (RCMAS-2): Manual.

[31] Al-Qerem W., Al-Maayah B., Ling J. (2021). Developing and Validating the Arabic Version of the Diabetes Quality of Life Questionnaire. East. Mediterr. Health J..

[32] The DCCT Research Group (1988). Reliability and Validity of a Diabetes Quality-of-Life Measure for the Diabetes Control and Complications Trial (DCCT). Diabetes Care.

[33] Kalra S., Jena B., Yeravdekar R. (2018). Emotional and Psychological Needs of People with Diabetes. Indian J. Endocrinol. Metab..

[34] Snoek R., van Jaarsveld R.H., Nguyen T.Q., Peters E.D.J., Elferink M.G., Ernst R.F., Rookmaaker M.B., Lilien M.R., Spierings E., Goldschmeding R. (2022). Genetics-First Approach Improves Diagnostics of ESKD Patients <50 Years Old. Nephrol. Dial. Transplant..

[35] Rechenberg K., Whittemore R., Grey M. (2017). Anxiety in Youth with Type 1 Diabetes. J. Pediatr. Nurs..

[36] Kovacs M., Mukerji P., Iyengar S., Drash A. (1996). Psychiatric Disorder and Metabolic Control Among Youths With IDDM: A Longitudinal Study. Diabetes Care.

[37] Northam E.A., Matthews L.K., Anderson P.J., Cameron F.J., Werther G.A. (2005). Psychiatric Morbidity and Health Outcome in Type 1 Diabetes—Perspectives from a Prospective Longitudinal Study. Diabet. Med..

[38] Jones J.M., Lawson M.L., Daneman D., Olmsted M.P., Rodin G. (2000). Eating Disorders in Adolescent Females with and without Type 1 Diabetes: Cross Sectional Study. BMJ.

[39] Hannonen R., Eklund K., Tolvanen A., Komulainen J., Riikonen R., Delamater A.M., Ahonen T. (2015). Psychological Distress of Children with Early-onset Type 1 Diabetes and Their Mothers’ Well-being. Acta Paediatr..

[40] Radobuljac M.D. (2013). Adolescent Risk Behavior Is Less Frequent in Patients with Type 1 Diabetes. J. Diabetes Metab..

[41] Sildorf S.M., Breinegaard N., Lindkvist E.B., Tolstrup J.S., Boisen K.A., Teilmann G.K., Skovgaard A.M., Svensson J. (2018). Poor Metabolic Control in Children and Adolescents with Type 1 Diabetes and Psychiatric Comorbidity. Diabetes Care.

[42] Young V., Eiser C., Johnson B., Brierley S., Epton T., Elliott J., Heller S. (2013). Eating Problems in Adolescents with Type 1 Diabetes: A Systematic Review with Meta-analysis. Diabet. Med..

[43] Bernstein C.M., Stockwell M.S., Gallagher M.P., Rosenthal S.L., Soren K. (2013). Mental Health Issues in Adolescents and Young Adults with Type 1 Diabetes. Clin. Pediatr..

[44] Eaton W.W., Mengel M., Mengel L., Larson D., Campbell R., Montague R.B. (1992). Psychosocial and Psychopathologic Influences on Management and Control of Insulin-Dependent Diabetes. Int. J. Psychiatry Med..

[45] Colton P.A., Olmsted M.P., Daneman D., Rodin G.M. (2013). Depression, Disturbed Eating Behavior, and Metabolic Control in Teenage Girls with Type 1 Diabetes. Pediatr. Diabetes.

[46] Engström I., Kroon M., Arvidsson C.-G., Segnestam K., Snellman K., Åman J. (1999). Eating Disorders in Adolescent Girls with Insulin-Dependent Diabetes Mellitus: A Population-Based Case-Control Study. Acta Paediatr..

[47] Anderson B.J., Laffel L.M., Domenger C., Danne T., Phillip M., Mazza C., Hanas R., Waldron S., Beck R.W., Calvi-Gries F. (2017). Factors Associated with Diabetes-Specific Health-Related Quality of Life in Youth with Type 1 Diabetes: The Global TEENs Study. Diabetes Care.

[48] Gross A.M., Delcher H.K., Snitzer J., Bianchi B., Epstein S. (1985). Personality Variables and Metabolic Control in Children with Diabetes. J. Genet. Psychol..

[49] Young-Hyman D., de Groot M., Hill-Briggs F., Gonzalez J.S., Hood K., Peyrot M. (2016). Psychosocial Care for People with Diabetes: A Position Statement of the American Diabetes Association. Diabetes Care.

[50] Smith M.S., Mauseth R., Palmer J.P., Pecoraro R., Wenet G. (1991). Glycosylated Hemoglobin and Psychological Adjustment in Adolescents with Diabetes. Adolescence.

[51] Lawler M.K., Volk R., Viviani N., Mengel M.B. (1990). Individual and Family Factors Impacting Diabetic Control in the Adolescent: A Preliminary Study. Matern. Child. Nurs. J..

[52] Melin E.O., Hillman M., Thunander M., Landin-Olsson M. (2019). Midnight Salivary Cortisol Secretion and the Use of Antidepressants Were Associated with Abdominal Obesity in Women with Type 1 Diabetes: A Cross Sectional Study. Diabetol. Metab. Syndr..

[53] Brossaud J., Corcuff J.-B., Vautier V., Bergeron A., Valade A., Lienhardt A., Moisan M.-P., Barat P. (2021). Altered Cortisol Metabolism Increases Nocturnal Cortisol Bioavailability in Prepubertal Children with Type 1 Diabetes Mellitus. Front. Endocrinol..

[54] Golden S.H. (2011). Emerging Therapeutic Approaches for the Management of Diabetes Mellitus and Macrovascular Complications. Am. J. Cardiol..

[55] Hinds J.A., Sanchez E.R. (2022). The Role of the Hypothalamus–Pituitary–Adrenal (HPA) Axis in Test-Induced Anxiety: Assessments, Physiological Responses, and Molecular Details. Stresses.

[56] Maguire A.M., Cowell C.T. (2007). Salivary Cortisol to Assess the Hypothalamic-Pituitary-Adrenal Axis in Healthy Children under 3 Years Old. J. Pediatr..

[57] Kristiansen E., Wanby P., Åkesson K., Blomstrand P., Brudin L., Thegerström J. (2020). Assessing Heart Rate Variability in Type 1 Diabetes Mellitus—Psychosocial Stress a Possible Confounder. Ann. Noninvasive Electrocardiol..

[58] Liu J.J.W., Ein N., Peck K., Huang V., Pruessner J.C., Vickers K. (2017). Sex Differences in Salivary Cortisol Reactivity to the Trier Social Stress Test (TSST): A Meta-Analysis. Psychoneuroendocrinology.

[59] Stroud L.R., Papandonatos G.D., Williamson D.E., Dahl R.E. (2011). Sex Differences in Cortisol Response to Corticotropin Releasing Hormone Challenge over Puberty: Pittsburgh Pediatric Neurobehavioral Studies. Psychoneuroendocrinology.

[60] Zeitoun M.H., Abdel Reheem A.A., Kharboush I.F., Sheshtawy H., Assad D.H., El Feky A.Y. (2023). Relationship between Depressive and Anxiety Symptoms and Fear of Hypoglycemia among Adolescents and Adults with Type 1 Diabetes Mellitus. Prim. Care Diabetes.

[61] Liakos A., Karagiannis T., Avgerinos I., Tsapas A., Bekiari E. (2023). Burden and Coping Strategies of Hypoglycemia in People with Diabetes. Curr. Diabetes Rev..

